# Construction of Layer-Blocked Covalent Organic Framework Heterogenous Films via Surface-Initiated Polycondensations with Strongly Enhanced Photocatalytic Properties

**DOI:** 10.1021/acscentsci.3c01195

**Published:** 2024-01-29

**Authors:** Yuxiang Zhao, Shengfei Li, Guangen Fu, Haoyong Yang, Shengxu Li, Daheng Wu, Tao Zhang

**Affiliations:** † Key Laboratory of Marine Materials and Related Technologies, Ningbo Institute of Materials Technology and Engineering, Chinese Academy of Sciences, Ningbo 315201, China; ‡ University of Chinese Academy of Sciences, Beijing 100049, China

## Abstract

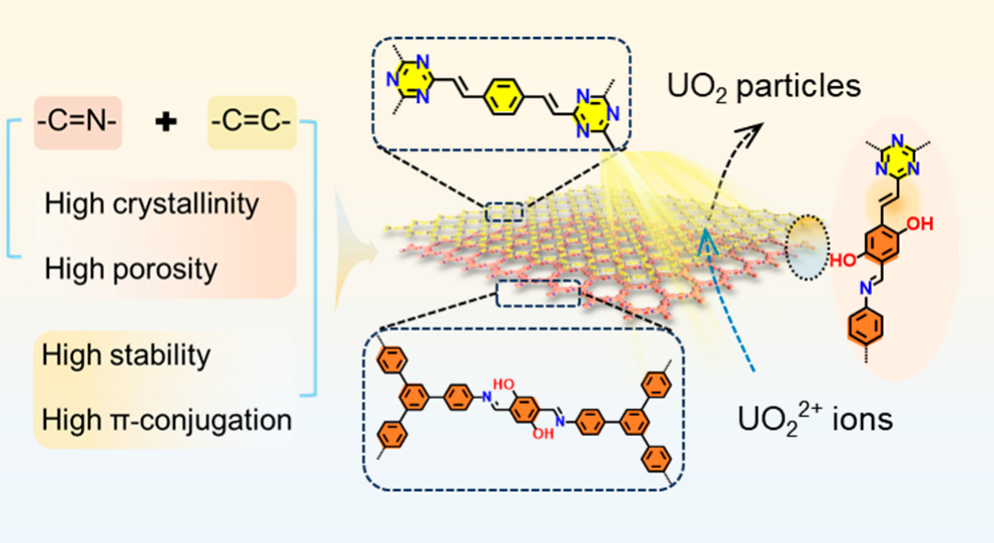

Imine-linked covalent organic frameworks (COFs) usually show high crystallinity and porosity, while vinyl-linked COFs have excellent semiconducting properties and stability. Therefore, achieving the advantages of imine- and vinyl-linkages in a single COF material is highly interesting but remains challenging. Herein, we demonstrate the fabrication of a layer-blocked COF (LB-COF) heterogeneous film that is composed of imine- and vinyl-linkages through two successive surface-initiated polycondensations. In brief, the bottom layer of imine-linked COF film was constructed on an amino-functionalized substrate via Schiff-base polycondensation, in which the unreacted aldehyde edges could be utilized for initiating aldol polycondensation to prepare the second layer of vinyl-linked COF film. The resultant LB-COF film displays excellent ordering due to the crystalline templating effect from the bottom imine-linked COF layer; meanwhile, the upper vinyl-linked COF layer could strongly enhance its stability and photocatalytic properties. The LB COF also presents superior performance in photocatalytic uranium extraction (320 mg g^–1^), which is higher than the imine-linked (35 mg g^–1^) and the vinyl-linked (295 mg g^–1^) counterpart. This study provides a novel surface-initiated strategy to synthesize layer-blocked COF heterogeneous films that combine the advantages of each building block.

## Abstract 2

A layer-blocked COF heterogeneous film, prepared via surface-initiated polycondensation, shared superior photocatalytic U(VI) extraction performance due to the formation of an S-scheme heterojunction.

## Introduction

Block copolymers (BCPs), in which variable chemically distinct segments connected by covalent bonds, are characterized by combining multiple functionalities in a single material.^1,2^ Since the emergence of BCPs in the 1950s, the feature that combines properties of multicomponent, especially amphiphilic, has been employed to prepare surfactants,^3^ micelles for drug delivery,^4^ as well as self-assembled nanostructures for electronics and catalysis.^2,5−7^ For example, the assembled BCP material composed of donor and acceptor chains is more conducive to enhancing the exciton dissociation and charge transfer performance for organic solar cells.^8^

Covalent organic frameworks (COFs) are crystalline porous polymers that can polymerize organic building blocks into extended network by covalent linkages,^9−11^ featuring well-defined extended framework, tunable functionalization and permanent porosity.^12,13^ Since the seminal progress reported by Yaghi et al. in 2005,^14^ various dynamic linkages with different reversibility, chemical stability and electron arrangement have been developed such as borate esters,^14^ imines,^15−17^ triazines^18,19^ and vinylene etc.^20−25^ Among them, the imine-linked COFs usually possess high crystallinity and porosity,^26−29^ but the hydrolysis and polarization of imine linkages limit the stability and charge carrier mobility. In another scenario, the vinyl-linked COFs exhibit excellent semiconducting property and stability due to its fully π-conjugated structures,^30,31^ providing superior performance for photocatalysis,^32−34^ photoluminescence^35−38^ and fluorescence detection.^39^

**Figure 1:**
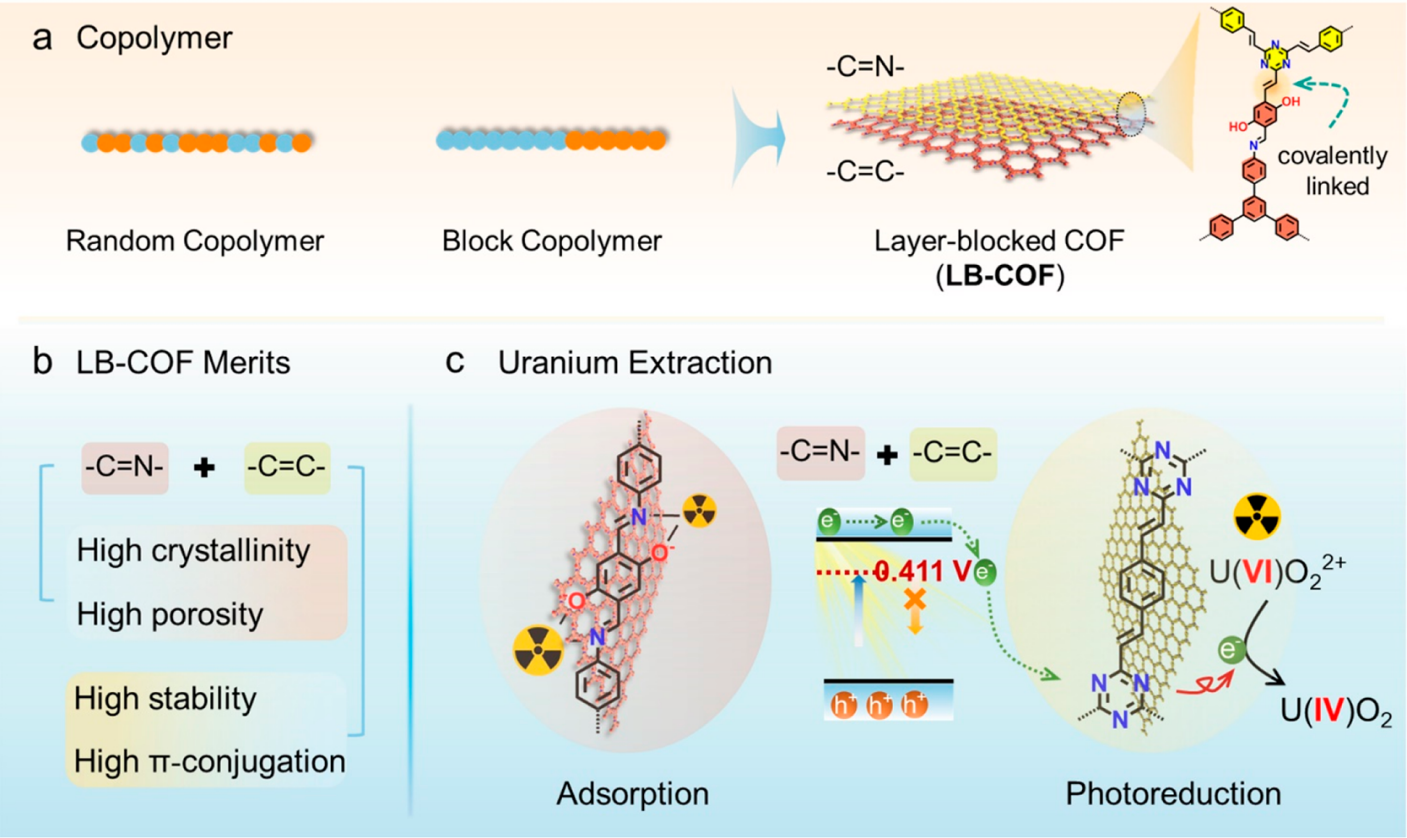
Design layer-blocked COF (LB-COF) inspired by the properties of classical BCPs. (a) Schematic diagram of BCPs and the LB-COF film. (b) The merits of LB-COF. (c) The uranium extraction process of LB-COF. Imine- and vinyl-linked COFs serve as adsorbent and photocatalyst, respectively.

Inspired by the advantages of classical BCPs, in this work, we show the fabrication of a layer-blocked COF (LB-COF) heterogeneous film through surface-initiated Schiff-base polycondensation and aldol polycondensation, respectively. The resultant LB-COF combines the advantages of high crystallinity of imine-linked COF and excellent photoelectric activity of vinyl-linked COF (Figure 1). The crystalline layer-blocked structure of the LB-COF was characterized by X-ray diffraction pattern (XRD), Fourier-transform infrared (FTIR) spectroscopy, and scanning electron microscopy (SEM). The enhanced photoelectric properties were demonstrated by photoelectrochemical, photoluminescence (PL), and surface potential analysis. Notably, LB-COF exhibited a photocurrent density up to 15 μA cm^–2^ at 0.3 V vs RHE, which is about 5 times in comparison to imine-linked COF (3 μA cm^–2^). As a photocatalyst for uranium extraction, the capacity of LB-COF (320 mg g^–1^) is significantly superior to those of the imine-linked (35 mg g^–1^) and vinyl-linked counterparts (295 mg g^–1^).

## Results and Discussion

### Synthesis and Characterizations of LB-COF Heterogeneous Film

**Figure 2:**
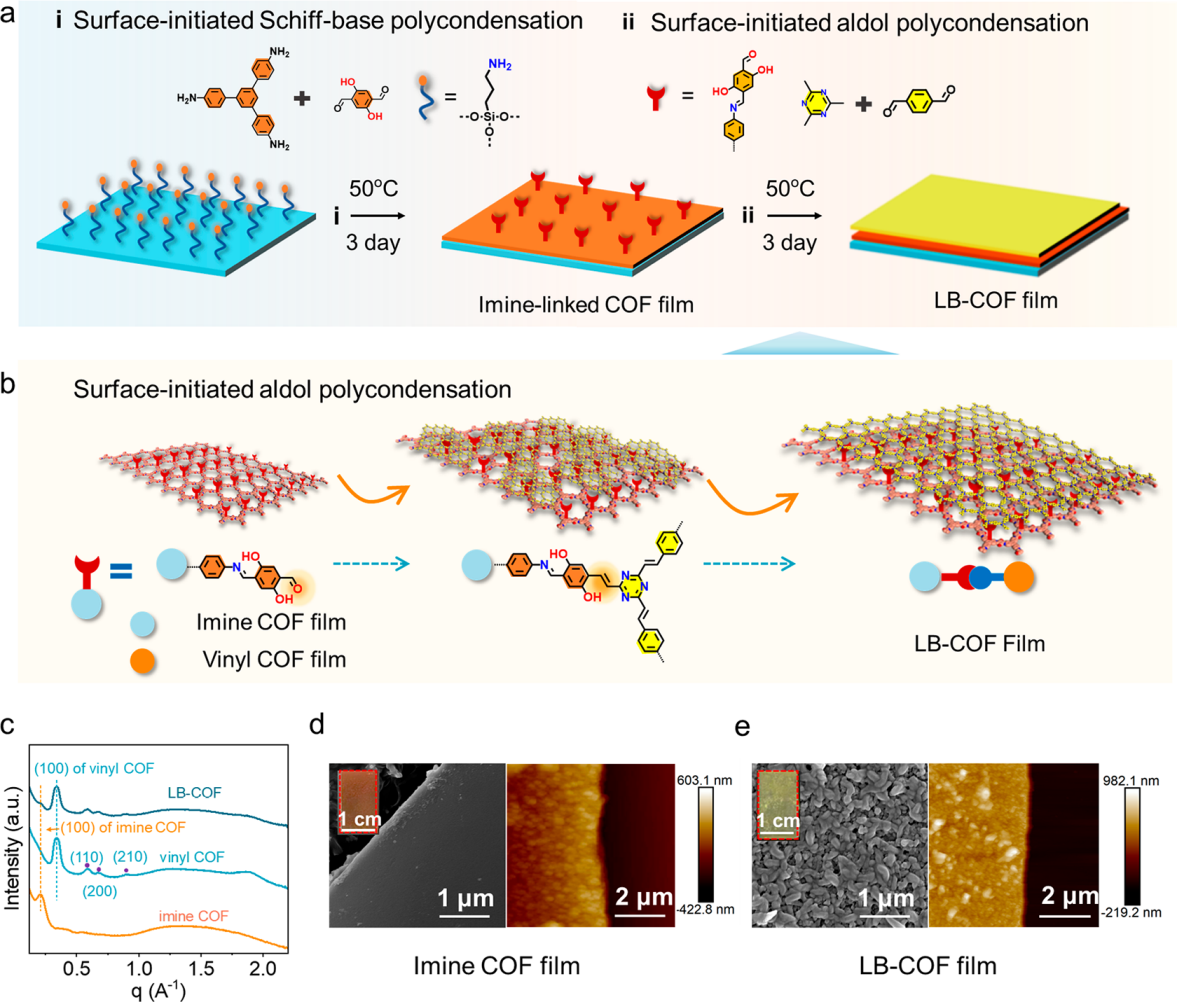
Synthesis and characterization of COF films. (a) Synthetic route to LB-COF heterogeneous film that is composed by imine- and vinyl-linkages through two successive surface-initiated polycondensations. (b) The unreacted aldehyde edges could be utilized to mediated aldol polycondensations. (c) PXRD patterns of COFs. (d,e) SEM and AFM images of imine-linked COF and LB-COF films.

As shown in Figure 2a, a planar silicon wafer covered with a self-assembled monolayer of initiator (3-aminopropyl-trimethoxysilane, APTES) was applied to initiate Schiff-base polycondensation. The formation of the APTES monolayer was demonstrated through X-ray photoelectron spectroscopy (XPS) (Supporting Information; Figure S1). The uniform imine-linked COF film was prepared by covalently linking monomers of 1,3,5-tris(4-aminophenyl)benzene and 2,5-dihydroxyterephthalaldehyde at liquid–solid interfaces, catalyzed by the scandium trifluoromethanesulfonate (Figure 2a).^40^ This film was subsequently placed into a solution of 2,4,6-trimethyl-1,3,5-triazine and 1,4-phthalaldehyde at 50 °C for 3 days, generating a uniform LB-COF film (Figure 2a).^41^ During the synthesis of LB-COF film, the unreacted aldehyde edges of imine-linked COF film were utilized to initiate aldol polycondensation (Figure 2b).

The grazing incidence wide-angle X-ray scattering (GIWAXS) pattern of the imine-linked COF demonstrates a hexagonal structure with an AA stacking mode and the oriented structure extends parallel to the substrate (Figure S2).^40^ The X-ray diffraction pattern (XRD) of yellow powders that exist in the reaction solution exhibits an obvious diffraction peak at 0.34 Å^–1^, completely corresponding to the (100) plane of vinyl-lined COF (Figure S3). The layer blocked structure (LB-COF) demonstrates two high peaks at 0.21 and 0.34 Å^–1^ that assign to the (100) planes of imine- and vinyl-linked COF films, respectively (Figure 2c). The poor reversibility of C=C bond weakens the error-correction process, which leads to relatively low crystallinity.^22,31^ Furthermore, the morphology of heterogeneous film was analyzed by scanning electron microscopy (SEM), atomic force microscopy (AFM) and transmission electron microscope (TEM) measurements. As illustrated in the SEM images (Figures 2d,e and S4), the surface of imine-linked COF is dense and smooth due to ordered extension parallel to the substrate, while vinyl-linked COF tends to form fibrous structures. Compared to imine-linked COF, the surface morphology transforms from smooth to fibrous after constructing the second layer of vinyl-linked COF (Figure 2d,e),^42^ and AFM images suggest the same conclusion (Figure 2d,e). TEM images reveal sheetlike structures at the nanoscale for both imine-linked COF and LB-COF (Figures S5 and S6).

The chemical structure of the COF films is identified by Fourier transformed infrared (FT-IR). In the spectrum of LB-COF (Figure S7), peaks at 1603 and 1632 cm^–1^ correspond to imine and vinyl linkages; meanwhile, the characteristic stretching band at 1664 cm^–1^ disappeared which indicates the unreacted aldehyde edges of imine-linked COF were utilized to mediate aldol polycondensation.^43−46^

### Optical and Electrochemical Properties

**Figure 3:**
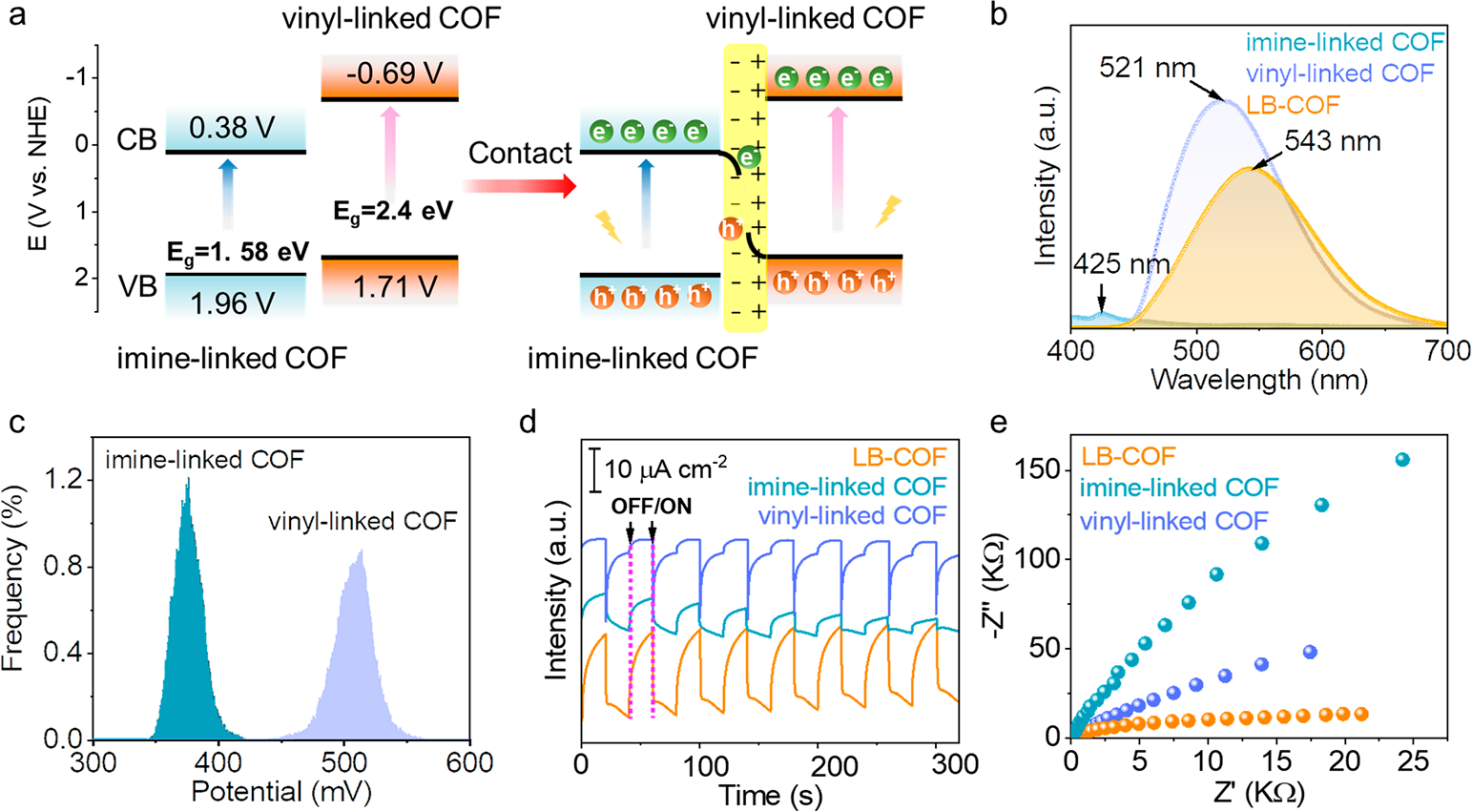
Photoelectronic properties of COF films. (a) Band structure diagram of LB-COF heterojunction. (b) Steady-state PL spectra of COFs. (c) Surface potentials of COFs. (d) Photocurrent–time plots of imine-linked COF, vinyl-linked COF and LB-COF films at 0.3 V vs RHE. (e) Impedance analyses of the COF films.

The electronic structures of COFs are characterized via the ultraviolet visible (UV–vis) absorption spectra and X-ray Photoelectron Spectroscopy (XPS) valence band spectrum. As shown in Figure S8, the optical band gaps of imine- and vinyl-linked COF are evaluated to be 1.58 and 2.4 eV according to Tauc plots. Combining with valence band (*E*_VB_) values (1.91 and 1.71 V vs NHE) determined via XPS valence band spectrum, we obtain the conduction band (*E*_CB_) values of 0.38 V and −0.69 V, based on the equation: *E*_CB_ = *E*_VB_ + *E*_g_ (Figure 3a). The photoluminescence (PL) and surface potential measurements further demonstrate that the layer-blocked COF forms an S-scheme heterojunction to promote the separation of photo generated electrons and holes. In the steady-state PL spectrum of LB-COF, upon excitation at 375 nm, the emission peak at 425 nm that belongs to imine-linked COF disappeared. Compared to pure vinyl-linked COF, the fluorescence intensity of LB-COF decreased and the emission peak wavelength shifted from 521 to 543 nm (Figure 3b), suggesting that generate electrons and holes can transfer between heterogeneous layers and a wider range of light can be utilized. The strong peak at 543 nm of LB-COF with a longer life indicates a higher density of photo generated electrons and holes in vinyl-linked COF compared to imine-linked COF (Figures S9 and S10). Additionally, the Kelvin probe force microscopy (KPFM) measurement uncovers that the surface potential of vinyl-linked COF is ca. 130 mV higher than that of imine-linked COF (Figure 3c). This represents a more negative Fermi level of vinyl-linked COF,^47^ and thus, charges will spontaneously migrate from vinyl-linked COF to imine-linked COF in terms of thermodynamic to form a built-in electric field when two layers of COF films are in close contact.^48^ The built-in electric field could promote the recombination of photo generated electrons from imine-linked COF and holes in vinyl-linked COF, forming an S-scheme heterojunction, which can significantly improve the photocatalytic performance of LB-COF. As expected, the LB-COF presents an outstanding photocatalytic performance. In photoelectrochemical (PEC) measurements, under visible-light irradiation, the photocurrent density of LB-COF film is up to 15 μA cm^–2^ at 0.3 V vs RHE, which is much larger than that of imine- and vinyl-linked COFs (3 and 5 μA cm^–2^) (Figure 3d). The electrochemical impedance spectroscopy (EIS) is also applied to measure the charge-transfer resistance. In the Nyquist plot (Figure 3e), the smaller diameter of LB-COF film reveals a higher charge transfer rate.

### Photocatalytic Uranium Extraction Studies

**Figure 4:**
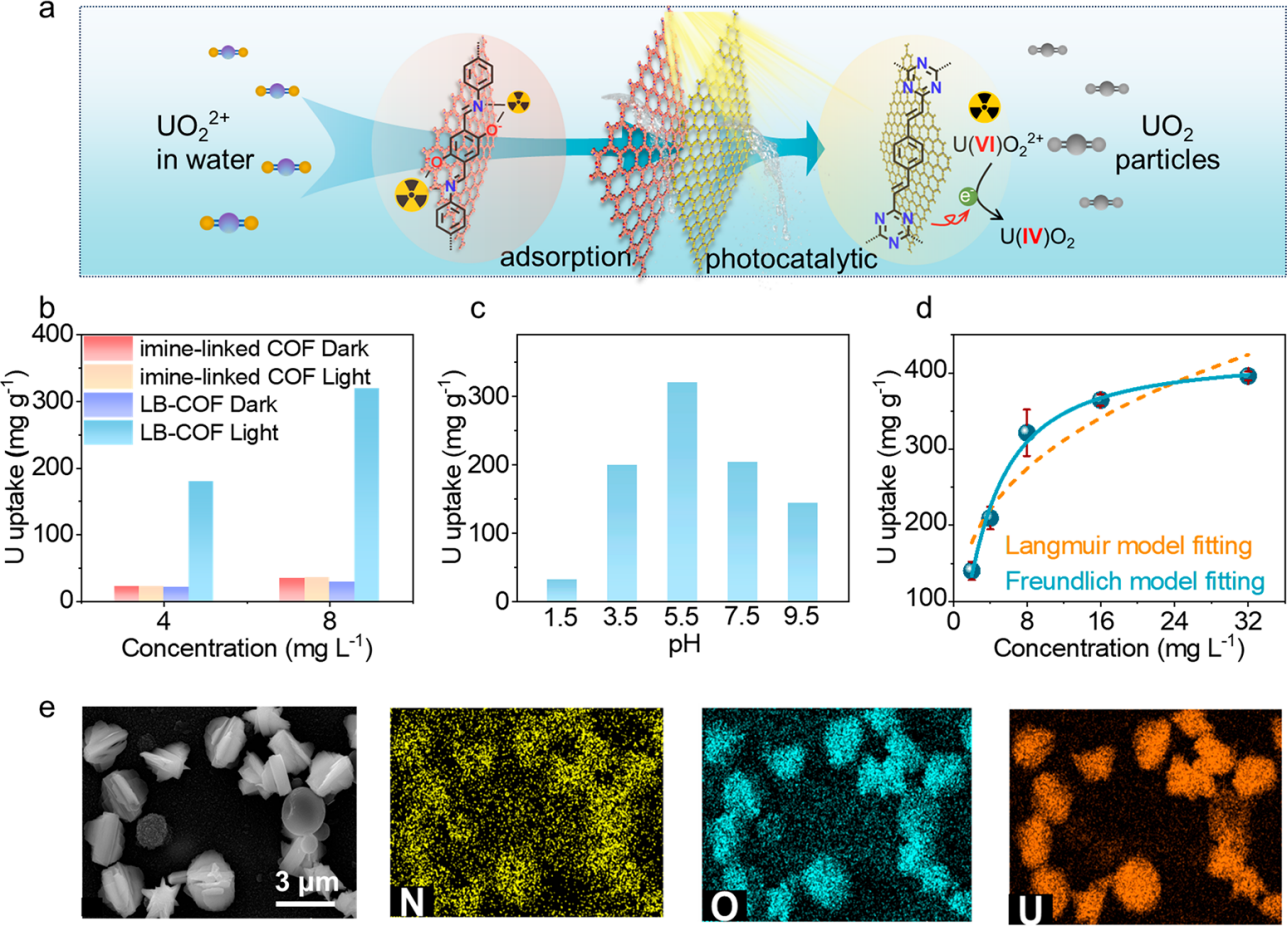
Photocatalytic uranium extraction performance. (a) Schematic diagram of photocatalytic UO_2_^2+^ reduction. (b) Uranium extraction capacity of COF films under light and dark conditions. (c) Dependence of uranium uptake on pH in 8 ppm of U-spiked water. (d) Equilibrium adsorption isotherms of LB-film and the corresponding fitting curves based on Langmuir model and Freundlich model under simulated sunlight irradiation. (e) EDS mapping of elements in the SEM image of U@LB-COF.

Photocatalytic uranium reduction is a vital technology for the utilization of marine uranium resources.^49−52^ The LB-COF with outstanding PEC performance and specific groups could work as an ideal photocatalytic uranium extraction material (Figure 4a). LB-COF film connected by covalent bonds possesses superior photocatalytic activity performance compared to the heterojunction film connected by noncovalent bonds due to the high efficient transport of charge carriers along the π-conjugated network. Significantly, from dark to visible-light (λ > 420 nm) conditions, the saturation capacities of LB-COF imine-linked sharply increased from 35 mg g^–1^ to ∼320 mg g^–1^. The value is higher than those of imine-linked COF (35 mg g^–1^) and vinyl-linked COF (295 mg g^–1^), at a concentration of 8 ppm (Figure 4b). Moreover, LB-COF proposes the optimal performance at a pH of 5.5 due to the protonation of the triazine nucleus (Figure 4c).^53^ The kinetic studies reveal that the equilibrium adsorption isotherm is more consistent with the Freundlich adsorption model, which evidence that photocatalytic uranium extraction is a chemical process^54^ (Figures 4d and S11). We measured the wavelength dependence of LB-COF for photocatalytic reaction upon different excitation wavelengths (405, 420, 455, 520, and 660 nm) (60 W·m^–2^) within 2 h (Figure S12). Upon the excitation of 420 nm, both imine- and vinyl-linked COF were excited; thus, the LB-COF exhibits higher photocatalytic uranium extraction capacity (170 mg g^–1^) than single imine- and vinyl-linked COF (35 and 152 mg g^–1^). The uranium extraction capacity of LB-COF decreased to 55 mg g^–1^ at 660 nm because only the imine-linked COF layer was excited.

**Figure 5:**
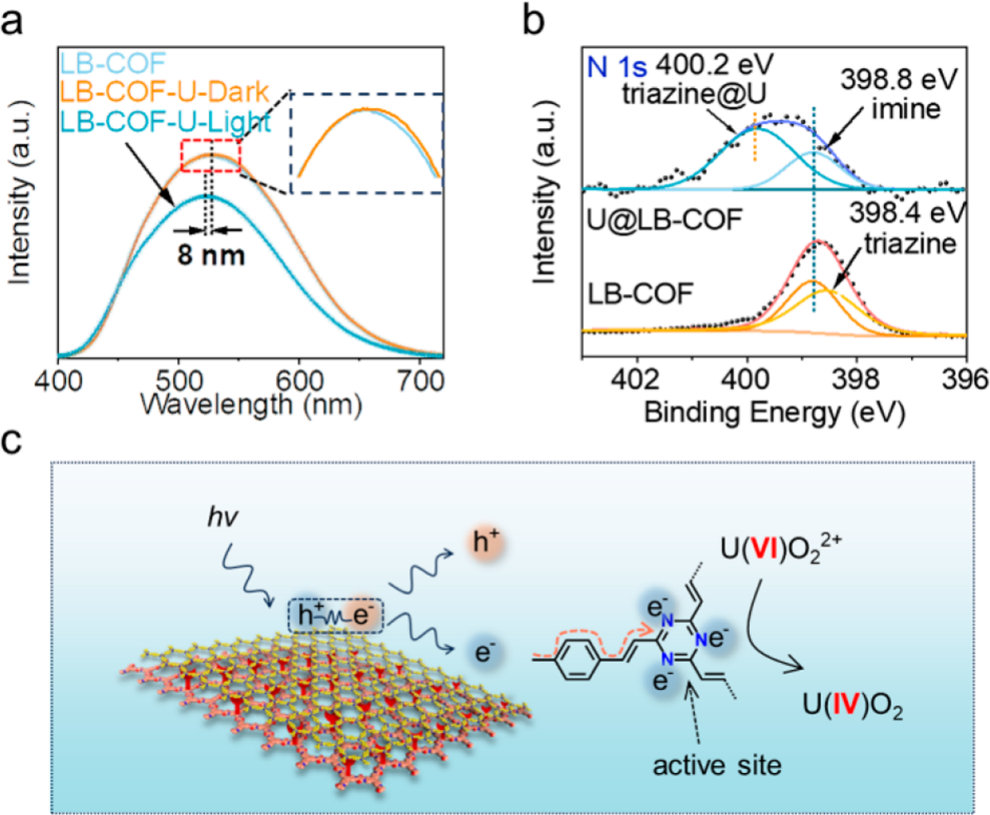
Interpretation of photocatalytic mechanisms. (a) Steady-state PL spectra of LB-COF and U@LB-COF. (b) High-resolution N 1s XPS spectra of LB-COF and U@LB-COF. (c) Schematic diagram of the photocatalytic uranium reduction mechanism.

To understand the conversion mechanism, we first verified the formation of UO_2_ via XPS spectra, which contains peaks of both U(VI)O_2_^2+^ and U(IV)O_2_ after uranium extraction (Figures S13 and S14).^55,56^ The SEM images of U@LB-COF also display uniform particles on the surface (Figure S15), which is considered as UO_2_ particles based on the analysis of the energy-dispersive X-ray (EDX) spectroscopy mapping (Figure 4e). The HRTEM image of U@LB-COF showed lattices of 0.31 nm corresponding to the (111) crystal face of UO_2_. The EDX spectroscopy mapping images suggest that U and O elements are uniformly distributed on the LB-COF film (Figure S16). The process of electron transfer is further explored via steady-state PL, in which the fluorescence intensity decreased when UO_2_^2+^ was added under illumination conditions, proving that photogenerated electrons are transferred to UO_2_^2+^ (Figure 5a). In this process, electron-withdrawing N atoms of triazine are considered to be active sites because the peak position in the N 1s XPS spectrum obviously shifted after uranium extraction (Figure 5b,c). The theoretical calculation also proves that photo generated electrons transfer from LB-COF to uranyl ions and N atoms on triazine are the electron transport active sites (Figure S17). Finally, LB-COF displays superb practical reusability, which still maintains 86% of the initial capacity after five cycles (Figure S18).

In summary, we present the construction of a layer-blocked COF heterogeneous film (LB-COF) that is composed by imine- and vinyl-linkages through two successive surface-initiated polycondensations. The resultant LB-COF film inherits the high crystallinity and superior photoelectric activity of imine- and vinyl-linked COFs, respectively. The LB-COF exhibits excellent photoelectrochemical performance (∼15 μA cm^–2^ at 0.3 V vs RHE), which is 5 times to imine-linked COF (∼3 μA cm^–2^) and 3 times to vinyl-linked COF (∼5 μA cm^–2^). The photocatalytic uranium extraction capacity of LB-COF (320 mg g^–1^) is also significantly higher than those of imine-linked COF (35 mg g^–1^) and vinyl-linked COF (295 mg g^–1^). This work provides a novel surface-initiated strategy to design layer-blocked COF films photocatalysts that combined the advantages of imine and vinyl linkages.

## Supporting Information Available

Materials, synthesis, characterization, and theoretical simulation details (PDF)
Transparent Peer Review report available (PDF)
