# Supplementary material for: Construction of Layer-Blocked Covalent Organic Framework Heterogenous Films via Surface-Initiated Polycondensations with Strongly Enhanced Photocatalytic Properties
Source: ACS Cent Sci. 2024 Jan 29;10(4):775–81. doi: 10.1021/acscentsci.3c01195 (PMC11046463; doi:10.1021/acscentsci.3c01195)
Supplement: Supplementary file 1 — oc3c01195_si_001.pdf [file oc3c01195_si_001.pdf]

Supporting Information

Construction of Layer-Blocked Covalent Organic Framework Heterogenous Films via Surface-Initiated Polycondensations with Strongly Enhanced Photocatalytic Properties

Yuxiang Zhao[1,2], Shengfei Li[1], Guangen Fu[1,2], Haoyong Yang[1,2], Shengxu Li[1,2], Daheng Wu[1,2], Tao Zhang[1,2*]

[1]Key Laboratory of Marine Materials and Related Technologies, Ningbo Institute of Materials Technology and Engineering, Chinese Academy of Sciences, Ningbo 315201, China

[2] University of Chinese Academy of Sciences, Beijing 100049, China

*Corresponding author. E-mail: tzhang@nimte.ac.cn

Supplementary Materials

Materials: Potassium hydroxide (90% AR), 1,3,5-tris(4-aminophenyl)benzene (TAPB) (97%), 2,5-dihydroxyterephthalaldehyde (95%), terephthalaldehyde (98%), ethyl acetate (99% AR), 1,2-dichlorobenzene (98%) and 1-butanol (99% AR) were purchased from Aladdin Biochemical Technology Co., Ltd. 2,4,6-trimethyl-1,3,5-triazine (TMT) (98%), scandium trifluoromethanesulfonate were purchased from Zesheng Technology Co., Ltd. Uranium nitrate (99%) and Arsenazo III (>95% AR) were purchased from Macklin Biochemical Co., Ltd. Unless otherwise stated, the commercially available reagents and solvents were used without further purification.

Supplementary Methods

Preparation of amino modified substrate

Silicon wafer with a nanoscale oxide layer (large-area flexible polyethylene terephthalate and curved polycarbonate glasses) was washed extensively with ethanol and deionized water for 5 min and dried by a jet of dry argon, then was treated by oxygen plasma (PEC-6, 600 W, sykejing company) for 10 min. Afterward, the substrates were placed in a closed container with a vial containing 30 μL of 3-aminopropyltrimethoxysilane. After the container was allowed to keep 80 ℃ for 2 h, the substrate was removed, washed with deionized water and ethanol then dried with a dry nitrogen stream[1].

Fabrication of imine-linked COF film

In a typical process, $Sc(CF_3SO_3)_3$ (3.9 mg, 0.008 mmol) as catalyst was dissolved in 10 mL water in a beaker (50 mL). Subsequently, 30 ml fresh ethyl acetate solution

dissolved with 1,3,5-tris(4-aminophenyl)-benzene (31 mg, 0.015 mmol) and 2,5-dihydroxy-1,4-benzenedicarboxaldehyde (14.9 mg, 0.0225 mmol) were transferred into the beaker. Then an amino modified substrate (5×5 cm) was placed in the solution and the beaker was sealed and kept without disturbance at 50°C for 3 days to form the imine-linked COF film on the substrate. Finally, the film was washed by ethyl acetate and ethanol three times to remove impurities[2].

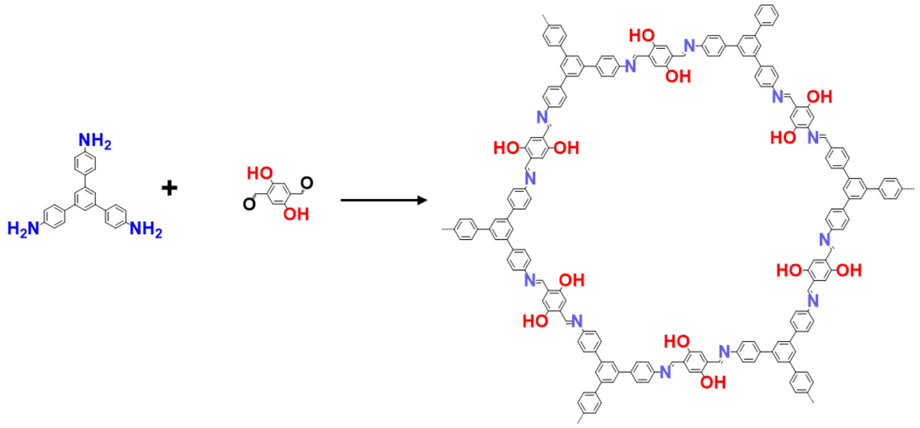

Scheme S1. Synthetic route towards imine-linked COF.

Fabrication of LB-COF film

KOH (80 mg) as catalyst was dissolved in 3 mL water in a beaker (20 mL). Subsequently, 10 ml mixed solution of o-dichlorobenzene (3 mL) and n-butanol (7 mL) dissolved with 2,4,6-trimethyl-1,3,5-triazine (TMT) (61.58 mg, 0.5 mmol) and terephthalaldehyde (100 mg, 0.75 mmol) were transferred into the beaker. Then the substrate with imine-linked COF film was placed in the solution and the beaker was sealed and kept without disturbance at 50°C for 3 days to form the LB-COF film. Finally, the film was washed by ethanol three times to remove impurities[3].

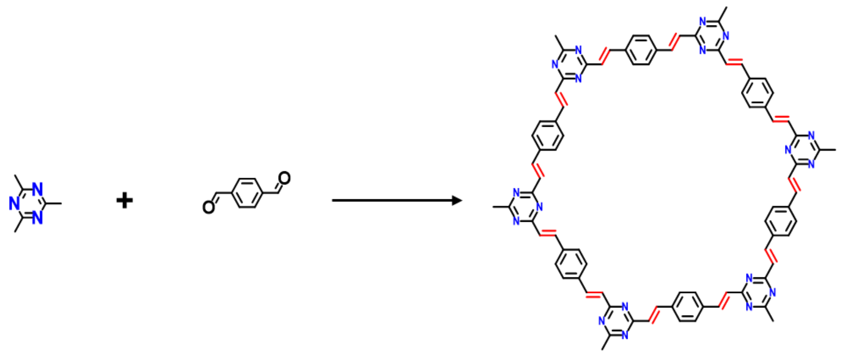

Scheme S2. Synthetic route towards vinyl-linked COF.

Characterization: Infrared spectra (IR) were recorded on a Thermo NICOLET 6700 Intelligent Fourier infrared spectrometer. X-ray photoelectron spectroscopy (XPS) was performed by using AXIS SUPRA spectrometer. The scanning electron microscope (SEM) images were acquired using Cold field high resolution scanning electron microscope. Atomic force microscopy (AFM) was performed by using Dimension ICON SPM. Transmission electron microscope (TEM) characterizations were conducted using Talos F200X with an accelerating voltage of 200 KV. Grazing incidence wide-angle X-ray scattering (GIWAXS) was performed at beamline BL16B1 of SSRF. The angle of incidence was 0.12° and the samples were exposed to the beam for 30 s. All samples are transferred onto silicon substrates before measurements. The x-ray diffraction pattern (XRD) was performed by using Xeuss 3.0 UHR. A Cu Ka X-ray source (GeniX3D Cu ULD), operating at 50 kV and 0.6 mA, produced radiation with a wavelength of 1.5418 Å.

Arsenazo III determination of uranyl

Arsenazo III method was employed to determine uranyl concentrations in U-spiked deionized water unless otherwise indicated. Uranyl solution was added to Arsenazo III mixing solution containing deionized water (3 mL), Arsenazo III (0.2 mL, 1 mM) and

HCl (0.8 mL, 0.1 M). After that, the absorbances at 652 nm were monitored by UV-Vis spectrophotometer[4].

Photocatalytic reduction of U(VI) in laboratory

To evaluate the photocatalytic reduction effect on the uranium adsorption capacity, A 300W Xe lamp with a light density of 1 kW/m^2 was utilized as the simulated sunlight. A dark field environment was used for the control experiment.

1 mg of COF adsorbent was added to 100 mL of U-spiked deionized water. The solution held under magnetic stirring for different times with the stirring speed of 300 r/min. At each time point, an aliquot was taken out and analyzed using Arsenazo III Assay. The uranium adsorbing capacity was calculated using the following equation:

$$q_t = (C_0 - C) \times \frac{V}{m} \qquad \text{(S1)}$$

where q_t is the amounts of adsorbed uranium at the exposure time (t); C_0 and C_t are the concentrations of uranium at time 0 and t, respectively; V is the volume of the solution (L) and m is the mass of the absorbent (g). At the equilibrium state, q_t and C_t are replaced by q_e and C_e, respectively.

Adsorption kinetic models and parameters

The pseudo-second-order and pseudo-first-order models were analyzed by the following equations.

$$\frac{t}{q_t} = \frac{1}{k_2 {q_e}^2} + \frac{t}{q_e} \qquad \text{(S2)}$$

$$\ln(q_e - q_t) = \ln q_e - k_1 t \qquad \text{(S3)}$$

where k_2 (g (mg min)$^{-1}$) and k_1 (min^{-1}) represent the corresponding rate constants.

PH-dependent uranium adsorption in U-spiked aqueous solution

1 mg of COF adsorbents were put into 100 mL of deionized water containing 8 ppm uranyl ions. The pH of the aqueous solution was adjusted by using sodium hydroxide and hydrochloric acid solution to 1.5, 3.5, 5.5, 7.5, 9.5, respectively. Uranium adsorption amount was calculated using Equation (S1).

Adsorption–desorption cycle test

Uranium laden samples were immersed into eluent composed of 1M Na_2CO_3 and 0.1 M H_2O_2 for the regeneration of adsorbents. Afterwards, aliquots of eluent were collected and analyzed via Arsenazo III assay for its uranium concentration. Thereafter, the desorption samples were rinsed with deionized water twice before being used for the next uranium sorption[5].

Density functional theory (DFT) calculations

The natural transition orbitals (NTOs) and the contributions of molecular orbital transitions were obtained by electron excitation analysis from the transition density matrix of TD-DFT calculation using Multiwfn program. The visualization of the natural transition orbitals used the Visual Molecular Dynamic program (VMD). From the NTOs, we can observe the spatial distributions of the hole and electron of the S1 excited state, with the hole being located on the LB-COF fragment whilst the electron was located at $[U(VI)O_2]^{2+}$ fragment, providing direct evidence of excited state charge transfer from the COF film to $[U(VI)O_2]^{2+}$.

Supplementary Figures

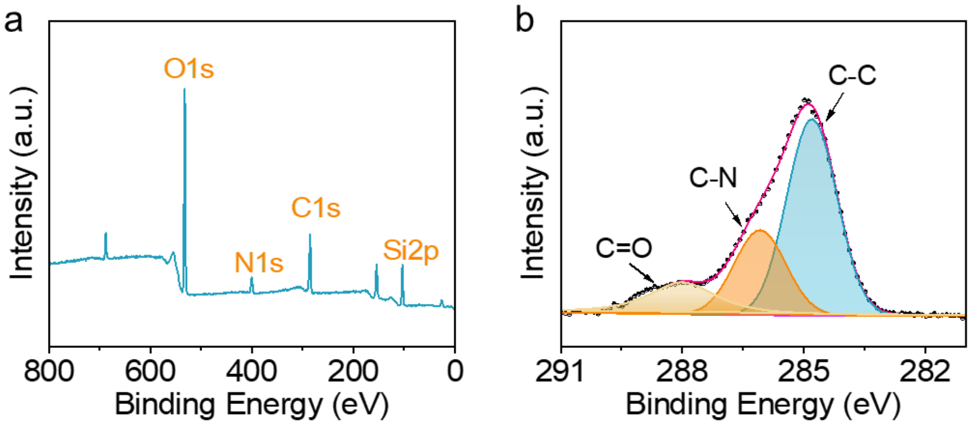

Figure S1. XPS spectra of APTES-functioned substrate. (a) XPS spectrum of APTES. The peak at ~400 eV corresponds to nitrogen element. (b) High-resolution spectrum of C1s of APTES. The peak at 286 eV corresponds to C-N of C-NH_2.

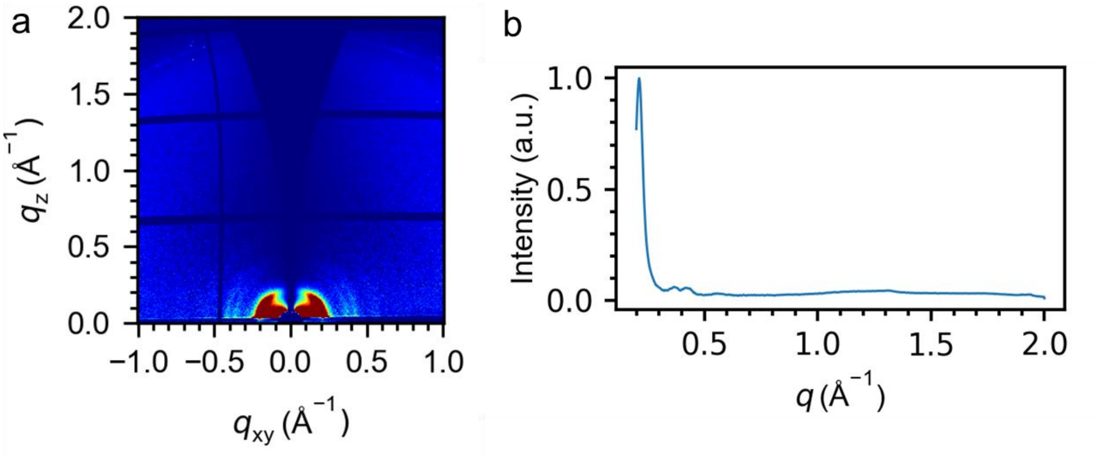

Figure S2. GIWAXS image of imine-linked COF, which demonstrates a highly oriented packing in the imine-linked film, where the imine-linked COF sheets are extended parallel to the substrate surface.

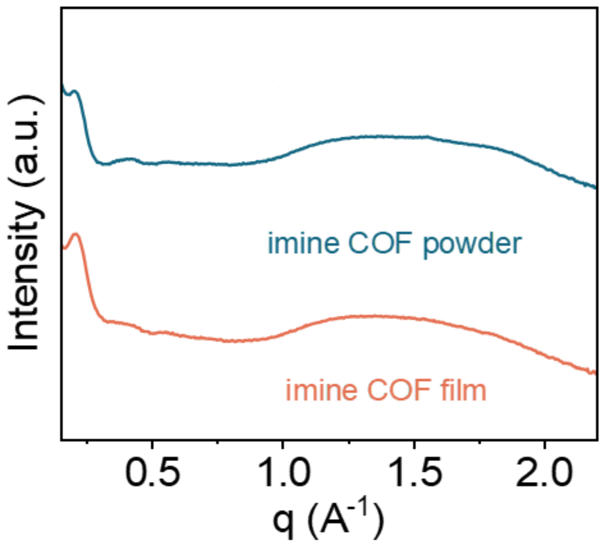

Figure S3. PXRD spectra of imine-linked COF powder and film.

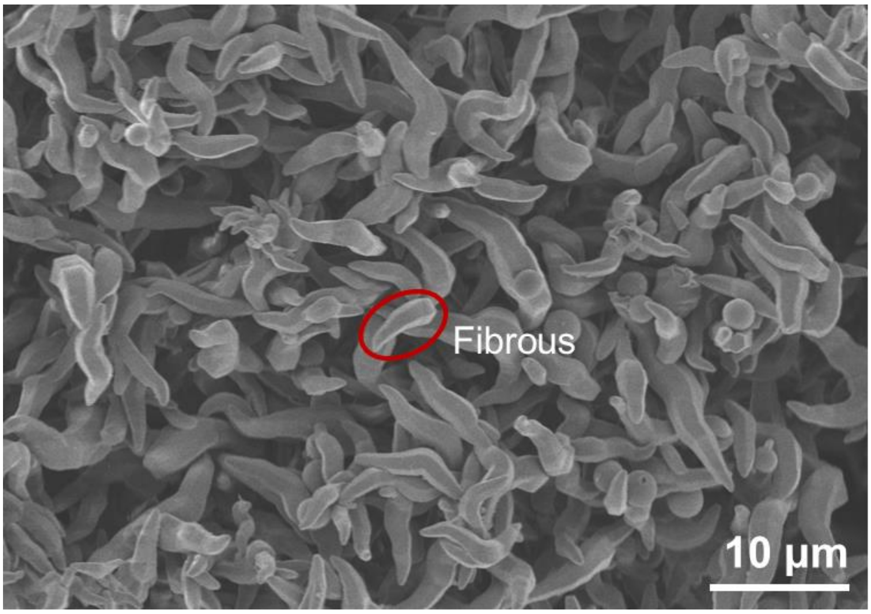

Figure S4. The SEM image of vinyl-linked COF powder proves that the morphology of vinyl-linked COF is fibrous, which corresponds to the morphology of the upper layer of the LB-COF.

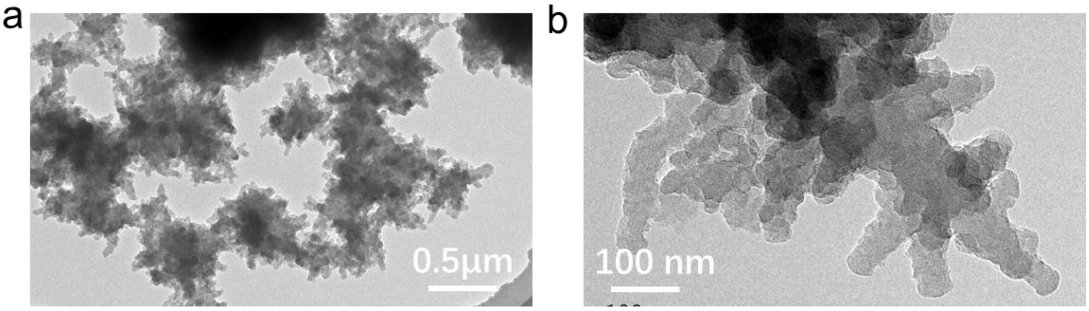

Figure S5. TEM images of imine-linked COF.

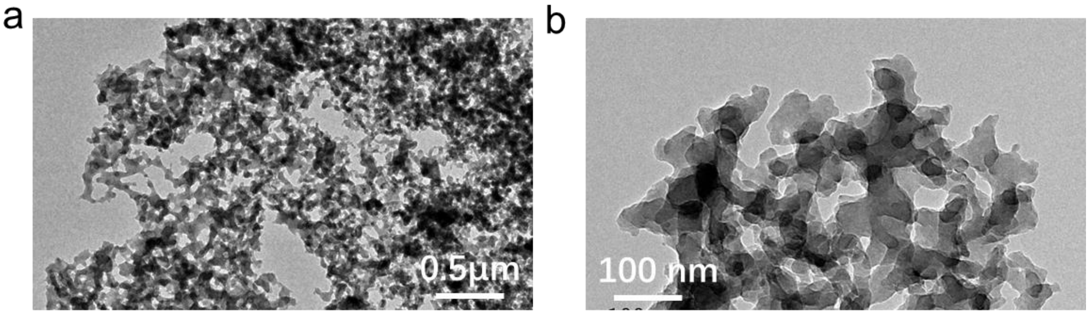

Figure S6. TEM images of LB-COF.

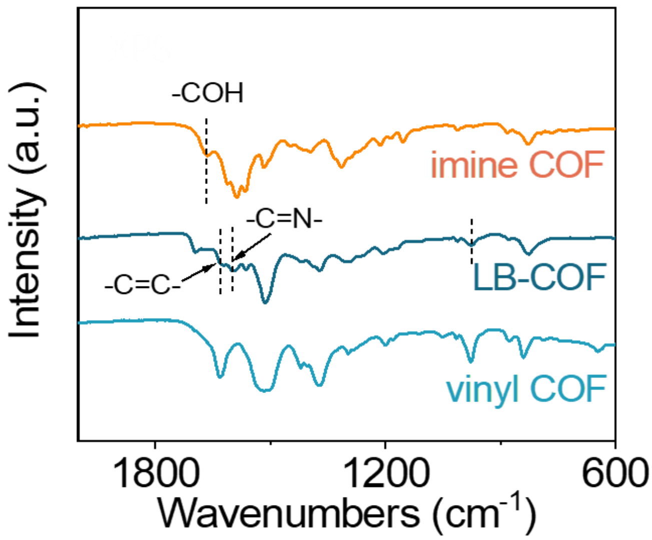

Figure S7. FT-IR spectra of imine-linked COF, vinyl-linked COF and LB-COF, which exhibits obvious peaks at 1603 and 1632 cm^{-1} assigned to C=N and C=C bonds, indicating the simultaneous existence of imine- and vinyl-linked COF linkages.

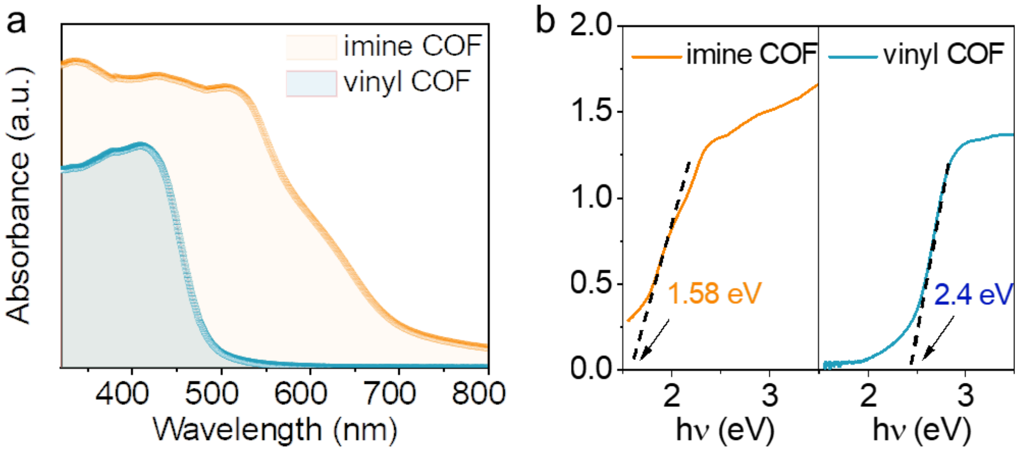

Figure S8. The band structure analysis of COF films. (a) Ultraviolet−visible (UV−vis) absorption spectroscopy of imine-linked COF (red) and vinyl-linked COF (blue). (b) Tauc plot of imine- and vinyl-linked COFs.

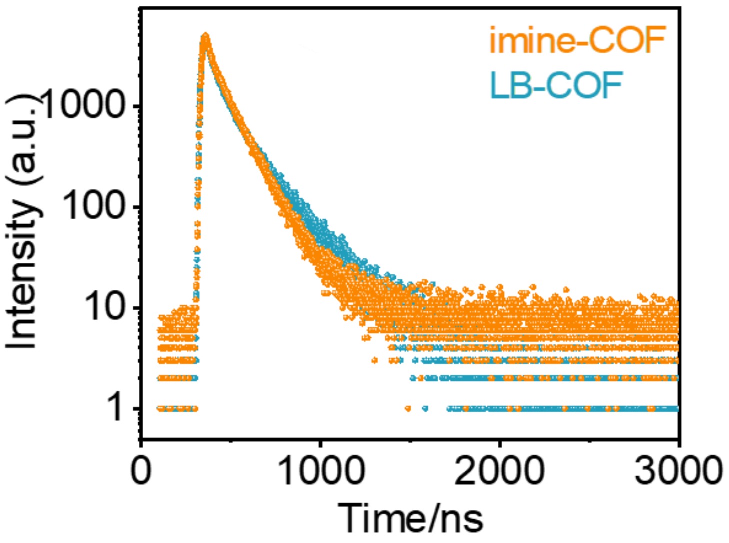

Figure S9. PL lifetime of imine-linked and LB-COF.

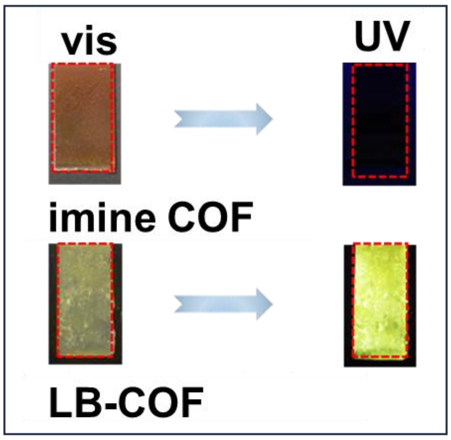

Figure S10. Images of imine-linked COF and LB-COF under 365 nm light and dark conditions. LB-COF exhibits bright yellow fluorescence, which is consistent with the test results of steady-state PL.

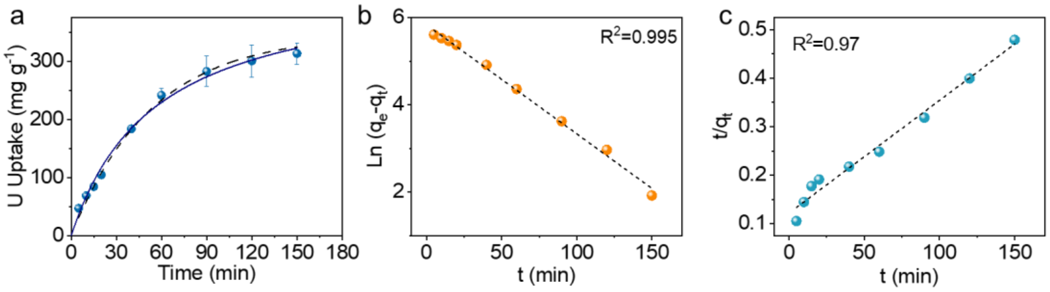

Figure S11. Adsorption kinetics in 8ppm uranium spiked aqueous solution. Pseudo-second-order model yields a better fit with a higher correlation coefficient (R^2) than the pseudo-first-order kinetic model, illustrating that uranium adsorption kinetics data can be better described by the pseudo-second order.

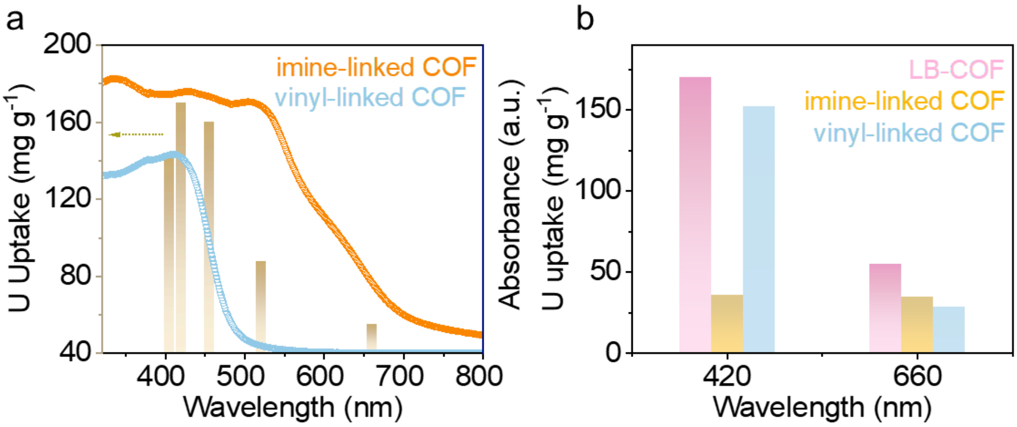

Figure S12. Wavelength dependence of LB-COF for photocatalytic uranium extraction.

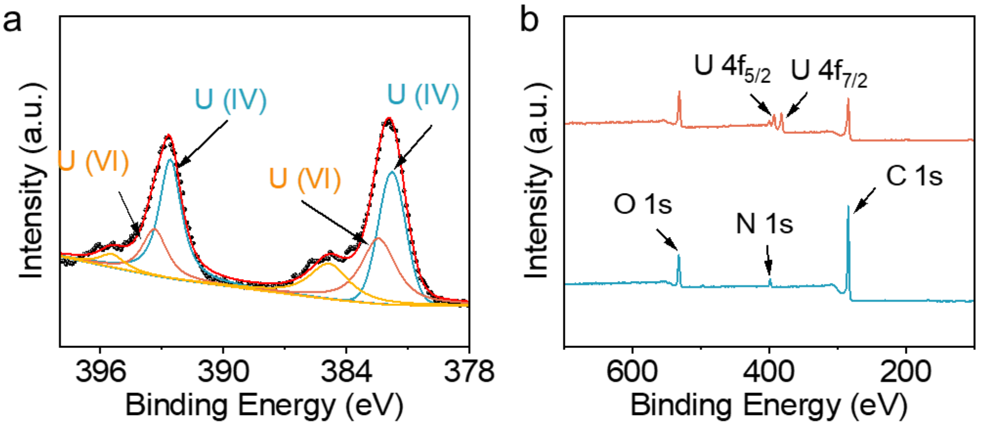

Figure S13. XPS spectra of imine-linked COF and U@imine-linked COF.

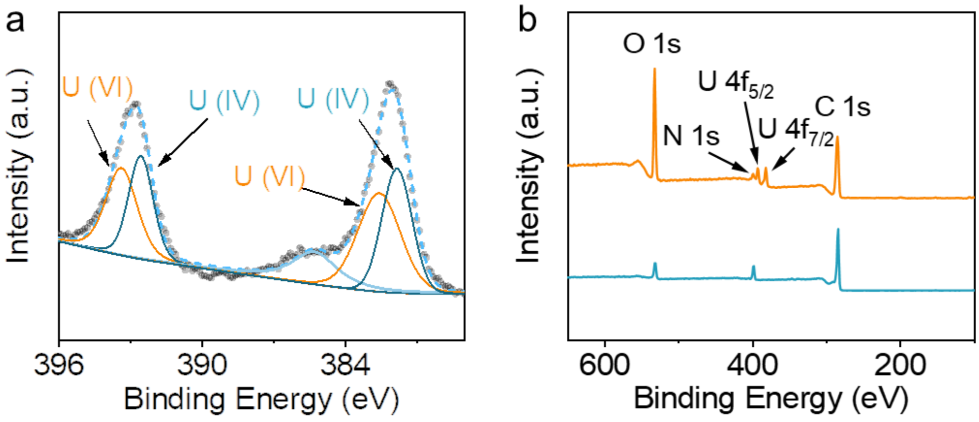

Figure S14. XPS spectra of LB-COF and U@LB-COF.

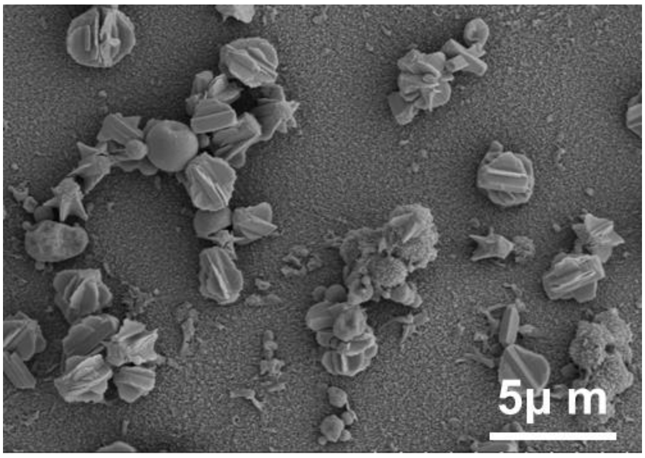

Figure S15. SEM image of LB-COF film after photocatalytic reduction of uranium. The particles are reduced uranium dioxide.

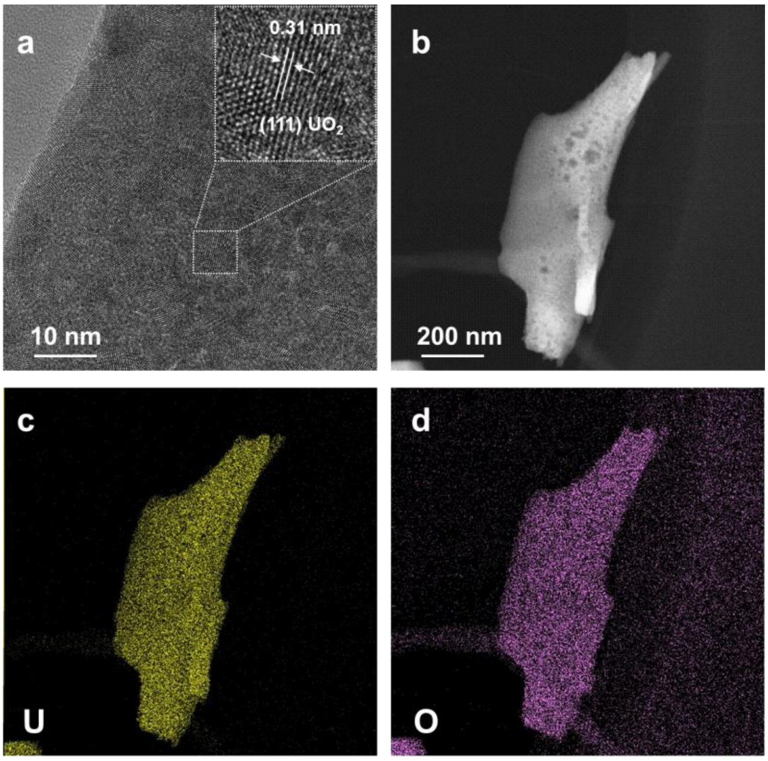

Figure S16. (a) TEM images of U@LB-COF. (b-d) EDX spectroscopy mapping of U and O elements in U@LB-COF.

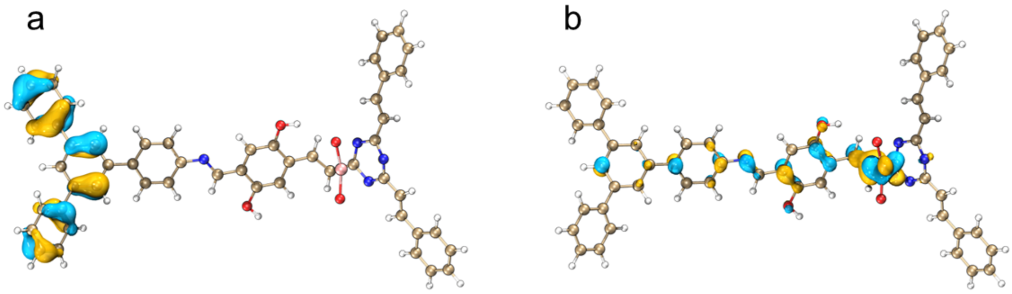

Figure S17. The excited state electronic structures of LB-COF photocatalysts (highlighting electron-hole distribution).

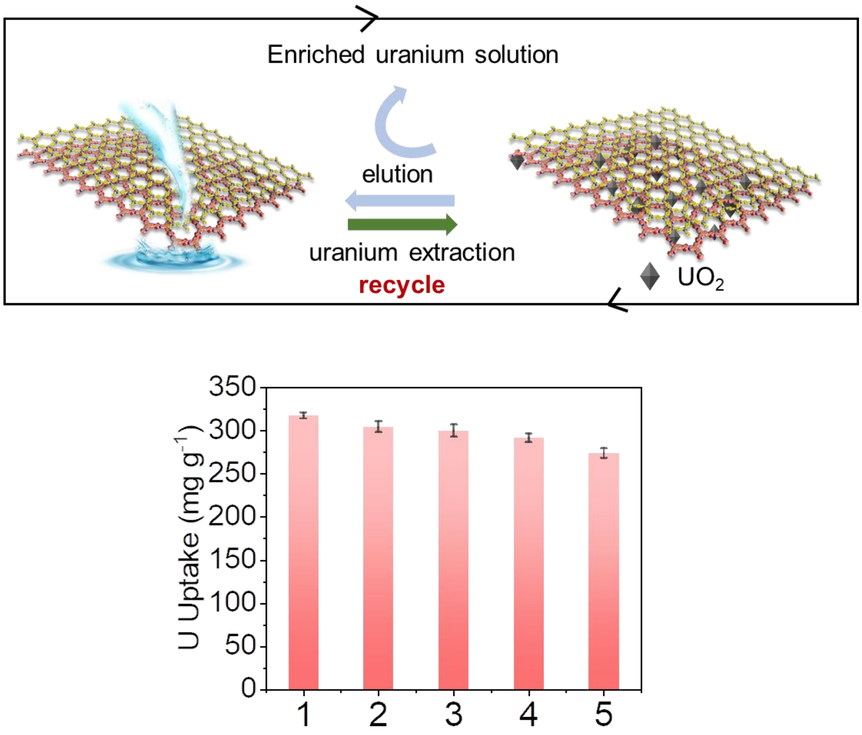

Figure S18. Performance of photocatalyst for uranium recovery. After five cycles, the LB-COF still maintained 86% of its initial uranium extraction capacity.

Reference

[1] X. Yin, D. Wu, H. Yang, J. Wang, R. Huang, T. Zheng, Q. Sun, T. Chen, L. Wang, T. Zhang, *ACS Macro Lett.* **2022**, *11*, 693-698.

[2] S. Zhao, C. Jiang, J. Fan, S. Hong, P. Mei, R. Yao, Y. Liu, S. Zhang, H. Li, H. Zhang, C. Sun, Z. Guo, P. Shao, Y. Zhu, J. Zhang, L. Guo, Y. Ma, J. Zhang, X. Feng, F. Wang, H. Wu, B. Wang, *Nat. Mater.* **2021**, *20*, 1551-1558.

[3] S. Wei, F. Zhang, W. Zhang, P. Qiang, K. Yu, X. Fu, D. Wu, S. Bi, F. Zhang, *J. Am. Chem. Soc.* **2019**, *141*, 14272-14279.

[4] M. Chen, T. Liu, X. Zhang, R. Zhang, S. Tang, Y. Yuan, Z. Xie, Y. Liu, H. Wang, K. V. Fedorovich, N. Wang, *Adv. Funct. Mater.* **2021**, *31*, 2100106.

[5] T. Liu, R. Zhang, M. Chen, Y. Liu, Z. Xie, S. Tang, Y. Yuan, N. Wang, *Adv. Funct. Mater.* **2021**, *32*, 2111049.
