# Supplementary material for: Construction of Layer-Blocked Covalent Organic Framework Heterogenous Films via Surface-Initiated Polycondensations with Strongly Enhanced Photocatalytic Properties
Source: ACS Cent Sci. 2024 Jan 29;10(4):775–81. doi: 10.1021/acscentsci.3c01195 (PMC11046463; doi:10.1021/acscentsci.3c01195)
Supplement: Supplementary file 2 — oc3c01195_si_003.pdf [file oc3c01195_si_003.pdf]

oc-2023-01195v.R1

Name: Peer Review Information for "Construction of Layer-Blocked Covalent Organic Framework Heterogenous Films via Surface-Initiated Polycondensations with Strongly Enhanced Photocatalytic Properties"

First Round of Reviewer Comments

Reviewer: 1

Comments to the Author

This paper describes the formation of COF LB film for photocatalytic application. The authors have developed COF-based photocatalysts.. The results can make our scientific knowledge largely developed. So, I recommend that this paper will be accepted after minor revisions about the points shown below.

1) Wavelength dependence for photcatalytic reaction should be measured. THe light with 420 nm of wavelength should exiceted both of the COF. If the authors excite one COF and compare the activity with the other, they can understand the mechanism more details.

2) Judging from the XRD patterns, the structure is more disordered than the image the authors show in the manuscript. I think they should be revisd for preventing misunderstanding.

Reviewer: 2

Comments to the Author

In this manuscript, Zhang and coworkers demonstrate the fabrication of a layer-blocked COF (LB-COF) heterogenous film that is composed by imine- and vinyl-linkages through two successive surface-initiated polycondensations. The resultant LB-COF combines the advantages of high crystallinity of imine-linked COF and excellent photoelectric activity of vinyl-linked COF. As a photocatalyst, the LB COF exhibits superior performance in uranium extraction with 320 mg g-1, which is much higher than the imine-

linked and the vinyl-linked counterparts. Overall, this study provides a novel and interesting surface-initiated strategy to synthesize layer-blocked COF heterogenous films that nicely combines the advantages of single building blocks. The manuscript is also well-organized and written. Thus, I strongly recommend this article to be published on ACS Central Science after minor revision.

1. The advantages of layer-blocked film connected by covalent bonds compared to the heterojunction film connected by noncovalent bonds should to be discussed in the revised manuscript to reflect the significance of this work.

2. The author indicated that self-assembled monolayer of initiator (3-aminopropyl-trimethoxysilane, APTES) can be applied to initiate Schiff-base polycondensation. Corresponding FT-IR and XPS characterizations should be supplied.

3. Imine-linked COF possessed wider light absorption range but exhibited poor photocatalytic uranium reduction performance. The authors need to provide a more detailed explanation.

4. The analysis of XPS spectra reveals that triazines are active sites for photocatalytic uranium reduction. Theoretical calculation should be supplemented to support the conclusion.

5. The authors demonstrate that photo generated electrons can transfer from imine-linked COF to vinyl-linked COF through the test of steady-state PL measurements. In order to reinforce the conclusion, the PL spectrum of pure imine- and vinyl-linked COFs should be supplied.

6. The authors should provide more evidence to prove that UO22+ was reduced to UO2.

7. The color style of Figures 3b-c is so different from other figures, which need to be uniformed.

Author's Response to Peer Review Comments:

We address the concerns of Reviewer 1# as follows:

Recommendation: Publish in ACS Central Science after minor revisions noted.

Comments:

This paper describes the formation of COF LB film for photocatalytic application. The authors have developed COF-based photocatalysts. The results can make our scientific knowledge largely developed. So, I recommend that this paper will be accepted after minor revisions about the points shown below.

Response:

We highly appreciate the constructive comments from the reviewer. The manuscript has been modified carefully according to the suggestions.

Question 1:

Wavelength dependence for photocatalytic reaction should be measured. The light with 420 nm of wavelength should excite both of the COF. If the authors excite one COF and compare the activity with the other, they can understand the mechanism more details.

Response:

Thanks for the constructive suggestion. Following the reviewer's suggestion, we measured the wavelength dependence of LB-COF for photocatalytic reaction upon different excitation wavelengths (405, 420, 455, 520 and 660 nm) (60 $W\cdot m^{-2}$) within 2h. Upon the excitation of 420 nm, both imine- and vinyl-linked COF were excited, thus the LB-COF exhibits higher photocatalytic uranium extraction capacity (170 mg g^{-1}) than single imine- and vinyl-linked COF (35 and 152 mg g^{-1}).

The uranium extraction capacity of LB-COF reduced to 55 mg g^{-1} at 660 nm because only the imine-linked COF layer was excited. Significantly, the value is higher than the single imine- and vinyl-linked COF (35 and 29 mg g^{-1}), suggesting that the internal electric field in S-scheme heterojunction drives the separation of electrons and holes (*Angew. Chem. Int. Ed.* **2022**, *61*, e202204108; *Chem* **2020**, *6*, 1543–1559).

Actions:

1. We add the **Figure R1-1** in **Figure S12 of the revised SI.**

2. We added the following discussion in **Page 5 of the revised manuscript**: "*We measured the wavelength dependence of LB-COF for photocatalytic reaction upon different excitation wavelengths (405, 420, 455, 520 and 660 nm) (60 W·m^{-2}) within 2h (Figure S12). Upon the excitation of 420 nm, both imine- and vinyl-linked COF were excited, thus the LB-COF exhibits higher photocatalytic uranium extraction capacity (170 mg g^{-1}) than single imine- or vinyl-linked COF (35 and 152 mg g^{-1}). The uranium extraction capacity of LB-COF reduced to 55 mg g^{-1} at 660 nm because only the imine-linked COF layer was excited.*"

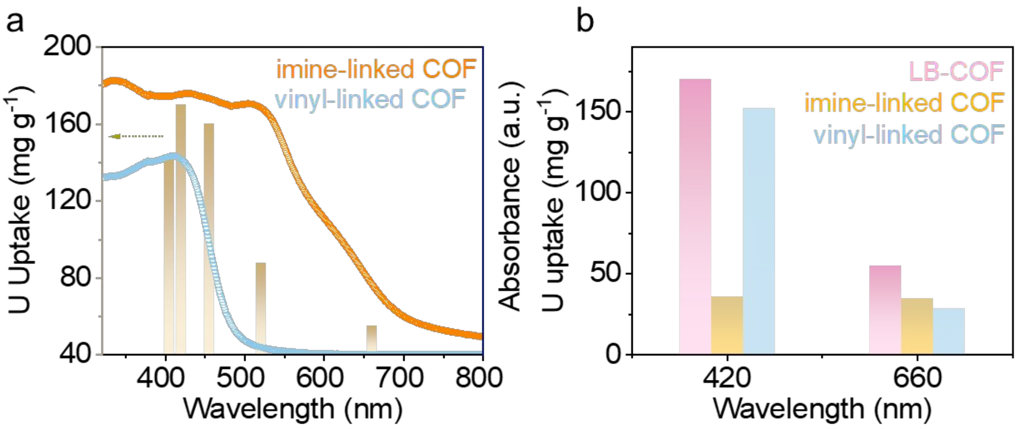

Figure R1-1. Wavelength dependence of LB-COF for photocatalytic uranium extraction.

Question 2:

Judging from the XRD patterns, the structure is more disordered than the image the authors show in the manuscript. I think they should be revised for preventing misunderstanding.

Response:

Thanks for your professional and constructive comments. We fully agree with the reviewer that the ordering of the LB-COF film is not very high. The reason is that the poor reversibility of C=C bond weakens the error-correction process, which leads to relatively low crystallinity. For such reason, the synthesis of highly ordered vinyl-linked COF has been quite challenging (*Polym. Chem*. **2016**, *7*, 4176-4181; *Angew. Chem. Int. Ed.* **2022**, *61*, e202111627; *Nat. Commun.* **2022**, *13*, 100; *Angew. Chem. Int. Ed*. **2022**, *61*, e202209762). For preventing misunderstanding, in the analysis of XRD patterns, we explain the reason for the low ordering of the LB-COF.

Actions:

1. We added the following discussion in **Page 2 of the revised manuscript**: "*The poor reversibility of C=C bond weakens the error-correction process, which leads to relatively low crystallinity.*"

We address the concerns of Reviewer 2# as follows:

Recommendation: Publish in ACS Central Science after minor revisions noted.

Comments:

In this manuscript, Zhang and coworkers demonstrate the fabrication of a layer-blocked COF (LB-COF) heterogenous film that is composed by imine- and vinyl-linkages through two successive surface-initiated polycondensations. The resultant LB-COF combines the advantages of high crystallinity of imine-linked COF and excellent photoelectric activity of vinyl-linked COF. As a photocatalyst, the LB COF exhibits superior performance in uranium extraction with 320 mg g^{-1}, which is much higher than the imine-linked and the vinyl-linked counterparts. Overall, this study provides a novel and interesting surface-initiated strategy to synthesize layer-blocked COF heterogenous films that nicely combines the advantages of single building blocks. The manuscript is also well-organized and written. Thus, I strongly recommend this article to be published on ACS Central Science after minor revision.

Response:

We highly appreciate the reviewer for the constructive comments. All the suggestions/comments from the reviewer have been carefully addressed and changes have been made accordingly.

Question 1:

The advantages of layer-blocked film connected by covalent bonds compared to the heterojunction film connected by noncovalent bonds should to be discussed in the revised manuscript to reflect the significance of this work.

Response:

Thanks for the helpful suggestions. Firstly, the covalently connected LB-COF heterogenous film possesses stronger interaction forces to avoid the separation of two layers in the photocatalytic process. Meanwhile, due to the high efficiently transport of charge carriers along the π-conjugated network (*Angew. Chem. Int. Ed.* **2023**, *62*, e202305978), the layer-blocked film prepared in this work possesses superior photo generated electron transfer and photocatalytic uranium extraction performance compared to the heterojunction film connected by noncovalent bonds.

Action:

We added the following discussion in **Page 5 of the revised manuscript**: "*LB-COF film connected by covalent bonds possesses superior photocatalytic activity performance compared to the heterojunction film connected by noncovalent bonds due to the high efficiently transport of charge carriers along the π-conjugated network.*"

Question 2:

The author indicated that self-assembled monolayer of initiator (3-aminopropyl-trimethoxysilane, APTES) can be applied to initiate Schiff-base polycondensation. Corresponding FT-IR and XPS characterizations should be supplied.

Response:

Thanks for the helpful suggestions. Due to the strong background effect of the silicon substrate, FT-IR spectrum did not show useful information of characteristic peaks. Instead, the XPS spectrum is more suitable for the characterization of APTES monolayer, which shows an obvious peak at ~400 eV corresponding N element and the high-resolution C1s spectrum exhibits a C-N peak at 286 eV, suggesting that the $-NH_2$ initiator was successfully modified on the surface of the silicon substrate.

Actions:

1. We add the **Figure R2-1** in **Figure S1 of the revised SI.**

2. We added the following discussion in **Page 2 of the revised manuscript:** "*The formation of APTES monolayer was demonstrated through X-ray photoelectron spectroscopy (XPS).*"

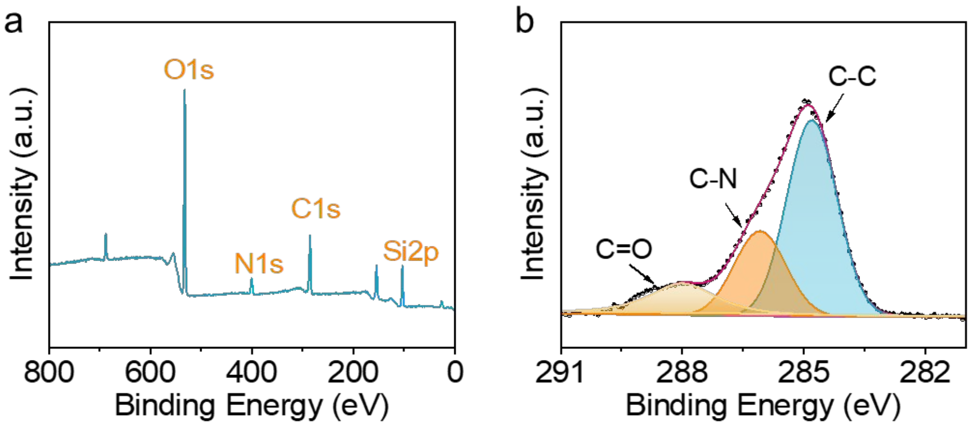

Figure R2-1. XPS spectra of APTES-functioned substrate. (a) XPS spectrum of APTES. The peak at ~400 eV corresponds to nitrogen element. (b) High-resolution spectrum of C1s of APTES. The peak at 286 eV corresponds to C-N of $-NH_2$.

Question 3:

Imine-linked COF possessed wider light absorption range but exhibited poor photocatalytic uranium reduction performance. The authors need to provide a more detailed explanation.

Response:

Thanks for your professional and constructive comments. The uranium reduction performance of photocatalyst is determined by the absorption ability of light, separation efficiency of photo generated charge carriers and the reduction ability of photogenerated electrons. Although imine-linked COF possessed wider light absorption range, a portion of energy was dissipated because of the vibration of imine bonds. Additionally, the conduction band value (0.38 V) of imine-linked COF is very close to the reduction potential of uranyl ions (0.411 V) (*Nat. Commun.* **2022**, *13*, 1389), indicating that conduction band electrons are difficult to drive uranium reduction thermodynamically owing to poor reduction ability. In contrast, the conduction band electrons of LB-COF with strong reduction ability are easier to drive the reduction of uranium.

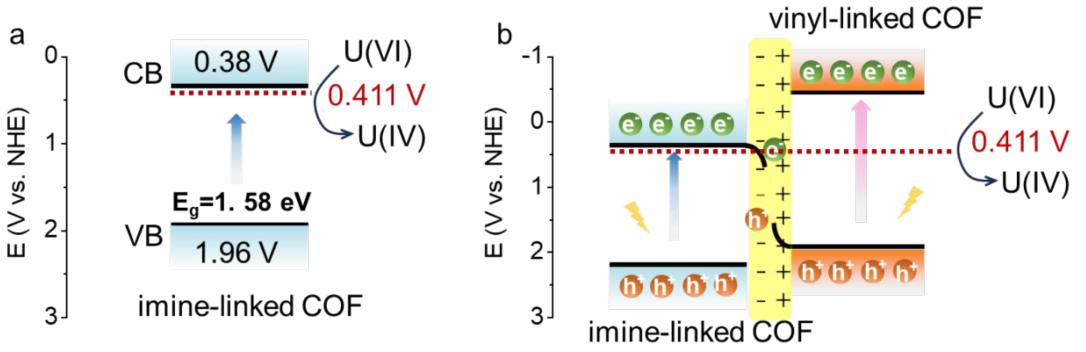

Figure R2-2. (a) Band structure diagram of imine-linked COF. The conduction band value is 0.38 V vs. NHE, close to the reduction potential of uranyl ions (0.411 eV). (b) Band structure diagram of LB-COF.

Question 4:

The analysis of XPS spectra reveals that triazines are active sites for photocatalytic uranium reduction. Theoretical calculation should be supplemented to support the conclusion.

Response:

Thanks for your professional and constructive comments. Regarding the reviewer's suggestion, we supplement the theoretical calculation, which shows the electronic distribution, electronic donation sites, and electron transport pathways. The electronic distribution on ground state and excited state reveal that photo generated electrons transfer from LB-COF to uranyl ions and N atoms on triazine is the electron transport active sites.

Actions:

1. We add the **Figure R2-3** in **Figure S17 of the revised SI.**

2. We added the following discussion in **Page 5 of the revised manuscript**: "*The theoretical calculation also proves that photo generated electrons transfer from LB-COF to uranyl ions and N atoms on triazine is the electron transport active sites (Figure S16).*"

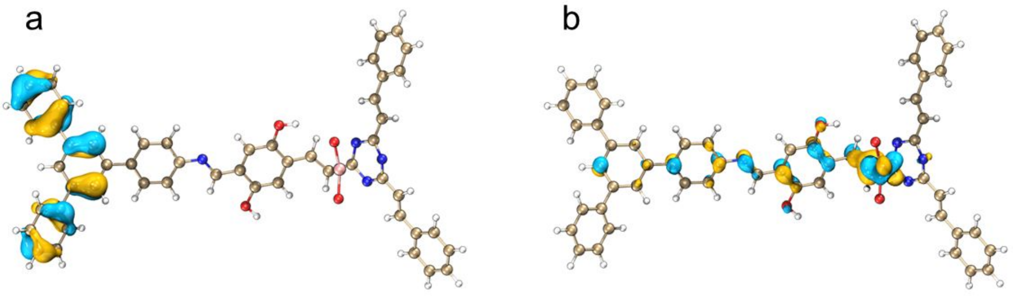

Figure R2-3. The excited state electronic structures of LB-COF photocatalysts (highlighting electron-hole distribution).

Question 5:

The authors demonstrate that photo generated electrons can transfer from imine-linked COF to vinyl-linked COF through the test of steady-state PL measurements. In order to reinforce the conclusion, the PL spectrum of pure imine- and vinyl-linked COFs should be supplied.

Response:

Regarding the reviewer's suggestion, the PL spectra of pure imine- and vinyl-linked COF have been supplied in the revised manuscript. Compared to pure vinyl-linked COF, the fluorescence intensity of LB-COF decreased and the emission peak wavelength shifted from 521 nm to 543 nm, revealing that a wider range of light can be utilized to excite COF for electrons generation, thus the photoelectrochemical properties is also enhanced.

Actions:

1. The new data and information have been added in **Figure 3b of revised manuscript**.

2. We added the following discussion in **Page 3 of the revised manuscript**: *"Compared to pure vinyl-linked COF, the fluorescence intensity of LB-COF decreased and the emission peak wavelength shifted from 521 nm to 543 nm (Figure 3b), suggesting that generate electrons and holes can transfer between heterogeneous layers and a wider range of light can be utilized."*

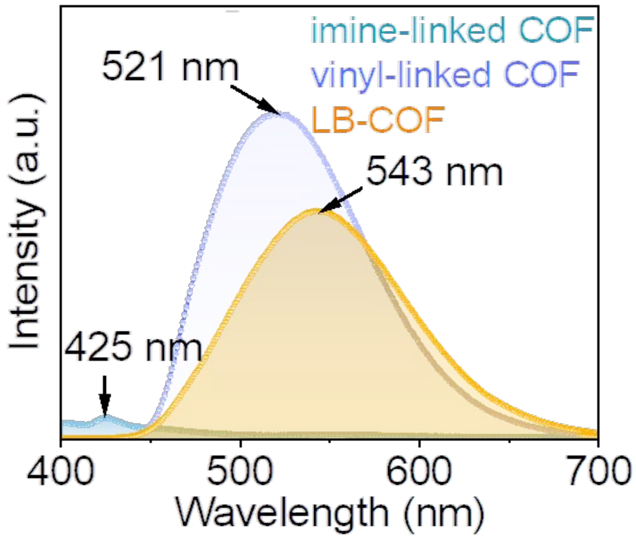

Figure R2-4. Steady-state PL spectra of imine-, vinyl-linked and LB-COFs.

Question 6:

The authors should provide more evidence to prove that UO_2^{2+} was reduced to UO_2.

Response:

Thanks for the constructive suggestions. Crystal structure of UO_2 was characterized by transmission electron microscopy (TEM). The HRTEM image of

U@LB-COF showed lattices of 0.31 nm corresponding to (111) crystal face of UO_2 (*Nat. Commun.* **2023**, *14*, 1106). The energy-dispersive X-ray (EDX) spectroscopy mapping images suggest that U and O elements are uniformly distributed on the LB-COF film.

Actions:

1. The new data and information have been added in **Figure S16 of revised SI**.

2. We added the following discussion in **Page 5 of the revised manuscript**: "*The HRTEM image of U@LB-COF showed lattices of 0.31 nm corresponding to (111) crystal face of UO_2. The energy-dispersive X-ray (EDX) spectroscopy mapping images suggest that U and O elements are uniformly distributed on the LB-COF film (Figure S15).*"

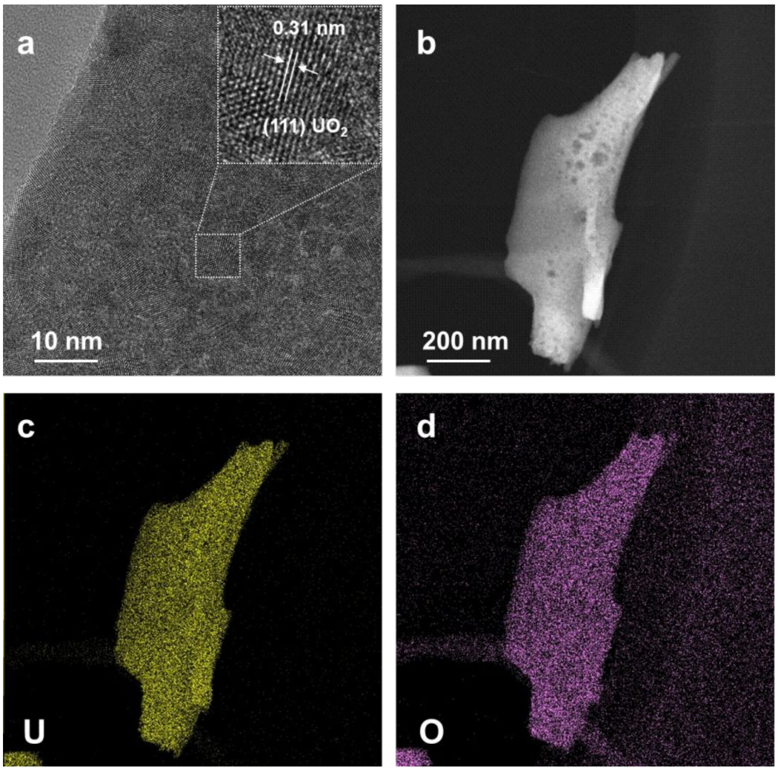

Figure R2-5. (a) HRTEM images of U@LB-COF. (b-d) EDX spectroscopy mapping of U and O elements in U@LB-COF.

Question 7:

The color style of Figures 3b-c is so different from other figures, which need to be uniformed.

Response:

We are sorry for these mistakes, which have been corrected in the revised manuscript. We further optimized the images in this manuscript to achieve a consistent style.
